# Evaluating Trends in Strangles Outbreaks Using Temperature and Precipitation Data in the United States of America for 2018–2022

**DOI:** 10.3390/pathogens12091106

**Published:** 2023-08-29

**Authors:** Bryce A. Thomas, Ryan K. Saylor, Zachary P. Taylor, DeLacy V. L. Rhodes

**Affiliations:** 1 Department of Biology, Berry College, Mount Berry, GA 30149, USA; bryce.thomas@vikings.berry.edu (B.A.T.); rsaylor@berry.edu (R.K.S.); 2 Department of Environmental Science and Studies, Berry College, Mount Berry, GA 30149, USA; ztaylor@berry.edu

**Keywords:** strangles, *Streptococcus equi* subspecies *equi*, horses, equids, GIS

## Abstract

Strangles is a highly contagious upper respiratory infection of equids that is globally distributed. The causative agent of strangles, *Streptococcus equi* subspecies *equi*, can be spread through indirect contact with infected fomites, and studies have shown this microbe to live well in varying environmental conditions. The purpose of this study was to analyze strangles case numbers across the United States of America from 2018 to 2022 to investigate potential temporal or weather patterns associated with outbreaks. Diagnosed case records were obtained from the Equine Disease Communication Center, university databases, government agencies, or veterinary diagnostic labs, and geographic information systems (GISs) were used to map cases and to acquire relevant meteorological data from outbreak areas. These data were analyzed using logistic regression to explore trends that occur between outbreaks and changes in temperature and precipitation. Initial review of weather data suggested monthly changes in strangles case numbers corresponded with changing seasons. Logistic regression indicated that changes in monthly average temperature and minimum temperature were significantly associated with increased or decreased odds of strangles outbreaks, respectively. Future analyses should focus on weather data isolated within a smaller region or state to better resolve trends in strangles outbreaks throughout the continental USA.

## 1. Introduction

Strangles is a highly contagious equine disease of the upper respiratory tract that is caused by *Streptococcus equi* subspecies *equi* (SEE). This disease can be found worldwide and is the third-most-common upper respiratory infection of horses in the United States of America (USA) [1], resulting in a high economic burden for horse owners [2]. Strangles is characterized by sudden-onset pyrexia, lethargy and pharyngitis, profuse mucopurulent nasal discharge, and lymphadenopathy with lymph node abscesses. In rare cases, these abscesses become so swollen that they rupture, disrupting breathing, hence the name of the disease [1,3]. Horses with strangles shed SEE in their nasal discharge, with approximately 10% of infected horses becoming subclinical carriers, harboring SEE in their guttural pouches after all signs of infection have cleared [3]. Treatment for strangles is controversial and not always performed due to the development of long-term immunity once an infection has been naturally cleared in 75% of infected horses [4]. When antibiotic therapy is used, SEE is typically treated with penicillin and is susceptible to most antibiotics [3].

Infection with SEE is spread through either direct contact with an infected horse or indirect transmission through fomites, a shared environment, or contact with shared human attendants [3,5]. Both environmental and direct contact transmission appear to be important in the spread and perpetuation of SEE. These bacteria have been shown experimentally to survive for extended periods of time in subfreezing temperatures, yet do not remain viable in hot, dry conditions [6]. Additionally, SEE has been found to persist on a variety of different surfaces but is typically inhibited by exposure to direct sunlight and can be cultured from environmental surfaces much longer during winter than summer months [6,7]. These studies demonstrate that local external conditions greatly affect the survivability of SEE, suggesting that environmental parameters such as temperature and precipitation could play an important role in the perpetuation of SEE in horse barns and in the occurrence of strangles outbreaks.

Surveillance of strangles outbreaks, active or passive, has occurred in limited studies within the USA. Currently, there are no mandatory surveillance systems in place for strangles, though it did become nationally monitored when it was added to the United States List of Reportable Animal Diseases (NLRAD) in 2017 [8]. Existing studies focused on the epidemiology of strangles have focused on identifying the genetic variability occurring between different SEE isolates, along with the different clinical manifestations observed during individual disease cases, and have quantified caseloads by month [1,9,10,11,12]. To date, no studies within the United States have sought to examine documented strangles outbreaks to look for patterns related to temporal and meteorological data. The continental United States is ideal for such a study because it contains a diverse range of climates. According to an updated Köppen–Geiger climate classification system [13], the US contains climates that range from tropical in southern Florida, subtropical in the eastern USA and along the west coast, continental or microthermal in the northern portions of the country, and extensive desert and steppe climates in the west. By studying strangles outbreak data that span the country, we can compare strangles occurrence across a wide range of weather conditions.

To explore any potential trends or patterns between temperature and precipitation and strangles outbreaks, we have acquired data on diagnosed strangles cases in the United States over a five-year period (2018–2022) and have used local temperature and precipitation data to determine if strangles outbreaks are affected by temperature and/or precipitation. The purpose of this study is to identify any existing patterns between weather conditions and cases of strangles in order to better understand the occurrence of these infections within the United States. An investigation of how weather conditions may relate to disease outbreaks will help veterinarians and horse owners to better understand patterns of strangles disease occurrences.

## 2. Materials and Methods

### 2.1. Outbreak Data Collection

**Figure 1:**
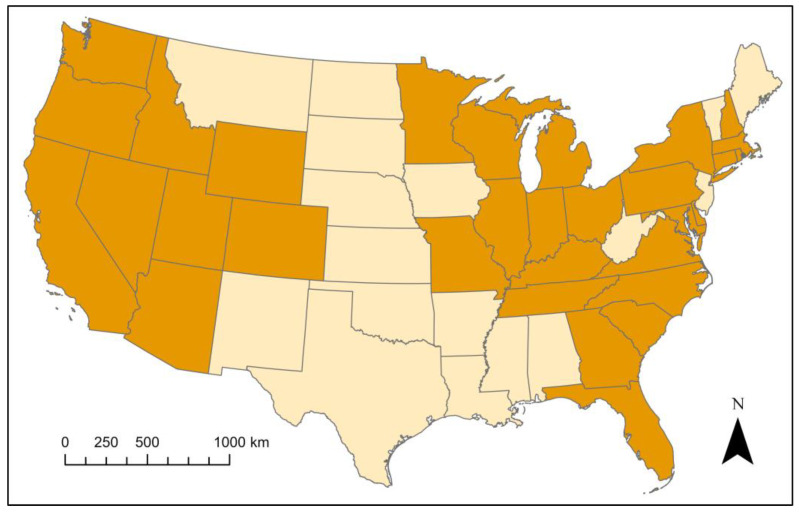
Map of the continental United States depicting states with reported data on strangles cases. States colored orange reported strangles data, while no data were collected from states colored cream. Alaska and Hawaii were not considered in this study.

Strangles occurrence data were acquired from multiple sources, such as the Equine Disease Communication Center [14] and University of Kentucky [15] online databases, the Georgia Department of Agriculture, the Wyoming State Veterinary Laboratory, the New York Department of Agriculture, the Montana Department of Livestock, and the Oklahoma Department of Agriculture, Food and Forestry. A total of 601 cases from 31 states were included in our analyses. In this study, the term ‘outbreak’ is used to describe at least a single horse being diagnosed with strangles during a particular time at a particular location. As there can be multiple horses on one barn infected with strangles at a time, we feel that the term outbreak is more inclusive than the term ‘case’. All data included in this study contain date and county of occurrence. Only cases that occurred between 1 January 2018 and 31 December 2022 were used in these analyses. Figure 1 shows which states reported strangles cases within the five-year study period. The number of cases reported by each state can be found in Table S1 and all case information, including county, date of occurrence, and weather data used in our analysis can be found in Table S2.

### 2.2. Temperature and Precipitation Data and GIS Analysis

We used temperature and precipitation data from the National Oceanic and Atmospheric Administration’s Monthly Gridded Dataset [16]. Using zonal statistic functions, we compiled the gridded temperature data into countywide mean values of maximum temperature, minimum temperature, mean temperature, and mean precipitation. We then joined the weather data to each outbreak using a concatenated key that denoted both the county and month of occurrence using ArcGIS Pro 3.03 (ESRI, Redlands, CA). All counties that did not report a single case in the given timeframe were omitted from the analyses. For this study, we used a standard meteorological definition of the seasons where spring includes the months of March, April, and May, summer includes June, July, and August, fall includes September, October, and November, and winter includes December, January, and February.

### 2.3. Statistical Analysis

Our analyses focused on logistic regression of outbreak occurrence (a binary predictor) against all continuous weather variables collected from all sites. We used a “nearest neighbor” approach to select a representative non-outbreak occurrence for the same month and site from the next calendar year. For example, if an outbreak occurred in March of 2019, the representative non-outbreak used for comparison was taken from March 2020 at the same site. Outbreaks that occurred in 2022 were paired with a non-outbreak occurrence from the previous year (e.g., 2021) at the same site and month. The combination of both methods was used to keep the data within our original 5-year study period and to include weather data associated with the notably higher frequency of outbreaks reported in 2021 and 2022. Average daily precipitation, maximum air temperature, minimum air temperature, and mean air temperature were used in the first logistic regression model to determine if baseline weather data were predictors of strangles outbreaks. The second regression included a new variable that was linked to the observed value compared to its month and site-specific 5-year average (hereafter referred to as “5-year average” data). For example, the 5-year average was subtracted from the observed values as an estimate of relative deviation from average conditions linked to each outbreak. The third regression included a new set of variables that incorporated how the original variables changed relative to the month prior to the outbreak being reported at each site (hereafter referred to as “month-to-month” data). More specifically, if the outbreak occurred in May 2019, the value observed in April 2019 was subtracted from that of May to determine the overall change in each parameter prior to the outbreak. The additional regressions were included because initial review of monthly outbreak frequency suggested outbreaks increased during seasonal transitions, i.e., spring to summer and fall to winter, when environmental variables would also be expected to change across all sites. The “corrplot” package and a correlation matrix was used to test for multicollinearity among all variables in this analysis. [17]. All logistic regression models were analyzed using a generalized linear model (*glm*) in the base statistics package available in R v.4.3.1 [18], while stepwise variable selection utilized the “*MASS*” package [19]. Akaike model selection criteria (AIC) and stepwise variable selection (both forward and backward) were used to help chose the best-fitting model if a significant regression was detected. All regression analyses assumed α = 0.05.

If a significant model was identified, additional analyses were employed to determine overall model performance because of the marked variation observed in weather data. The predictive ability of the model was assessed using a train to test data ratio of 80:20 using the “*car*” [20] and “*tidyverse*” [21] packages, and the area under the curve (AUC) scores from receiver-operating curves (ROCs) were determined using the “*ROCR*” package [22]. The AUC values fall between 0 and 1 such that models with values close to unity (but at least ≥0.7) would indicate the chosen model adequately discriminated outbreaks from non-outbreak conditions [23]. The same significant model was reanalyzed by randomly selecting a new 80:20 (train to test) data set that contained the same variables. Each new data set was reanalyzed using logistic regression and model significance and performance were assessed a total of 100 times. Random remodeling was used to assess how many logistic models were significant out of 100 total models analyzed. Model coefficients, odds ratios with lower and upper confidence intervals, and AUC scores were reported as averages for all significant models detected from these analyses. Finally, bootstrap resampling with replacement using the same 80% ratio was performed using the “*boot*” [24] package to provide a better estimate of each logistic regression model parameter and its confidence interval. A total of 1000 random bootstrap resampling events and concurrent logistic regressions were performed on the data. Model coefficients and odds ratios with lower and upper confidence intervals were also reported as average values of the 1000 bootstrap events. Bootstrap resampling was included in the analysis because it does not assume any distribution (e.g., is nonparametric) among weather data, but random resampling provided a method for measuring the accuracy of parameter estimates linked with the logistic regression models. Use of both parametric and nonparametric methods to estimate model parameters ensured our estimates were an accurate reflection of the weather data and strangles outbreaks. The random logistic models were assessed assuming α = 0.05, but we also assessed α = 0.10 to account for variation in weather data, while 95% confidence intervals were calculated for each model parameter using the “boot.ci” function.

## 3. Results

### 3.1. General Trends in Outbreak and Weather Data

**Figure 2:**
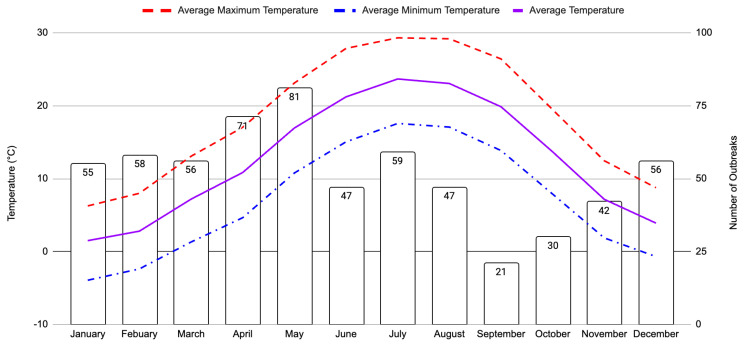
Representation of total number of strangles outbreaks shown with air temperatures. The total number of outbreaks per month across the five-year period is represented by a bar graph. The number inside each bar indicates the total number of cases reported for each month. Temperature data are represented as a line graph with average maximum temperatures shown as a red dashed line, average minimum temperatures as a blue dashed and dotted line, and average temperatures are represented by the purple solid line. The left *y*-axis denotes temperature (°C), the right *y*-axis shows the number of outbreaks, and the *x*-axis is labeled by month.

Temperature change from one month to another may affect the frequency of strangles outbreaks throughout the continental USA (Figure 2). The greatest positive rate of temperature change occurred between April and May, when average temperature increased by 6.1 °C, whereas the greatest negative rate of temperature change of nearly 6.5 °C occurred between October to November and November to December. Months with the highest strangles outbreaks were also linked with average air temperatures that were greater than 4 °C but less than 17 °C. Strangles outbreaks were most frequent in the month of May, followed by a decline over the summer months during peak temperatures, and with the lowest incidence of strangles occurring in September (Figure 2). The frequency of outbreaks then increased until December, where outbreak occurrence remained similar until increasing again in April and reaching its peak in May. Increases in the frequency of strangles outbreaks were also observed during months with (1) a near 5 °C decrease in month-to-month air temperature between September and December and (2) a nearly 5 °C increase in month-to-month air temperature between March and May.

All three temperature variables follow the same trends associated with North American seasons. The times in which temperature is increasing or decreasing at the greatest rate correspond with the maximum and minimum outbreak frequencies (Figure 2). When the temperature is at the greatest, lowest, or with a lower rate of change, outbreak frequency also remained more or less consistent during these months, i.e., December to March and June to August (Figure 2). For example, rates of temperature change between these remaining months were all generally much less than 3 °C and average air temperature was near freezing or remained above 20 °C. Precipitation was also explored in this study, yet no discernable trend was observed, which suggests there is little change in the rate of precipitation and no obvious link to outbreak frequency over the course of the five-year study period (Figure S1).

### 3.2. Logistic Regressions

One of the three initial logistic regressions of weather data was found to contain significant predictors of strangles outbreaks. The model that included the month-to-month weather variables was found to be a significant predictor of strangles outbreaks (Table 1). The models containing the observed and 5-year average variables were not significant and multicollinearity was high between all observed temperature variables in model one and was also detected between the 5-year average temperature and 5-year minimum temperature of model two. Stepwise variable selection and AIC indicated that (month-to-month) minimum temperature and average temperature were the only significant predictors of outbreak in the most parsimonious model (AIC = 1666.3) and only moderate correlation was detected between these variables in the final model. The latter was true for any model that included minimum air temperature, regardless of its significance, because the AIC score increased when minimum air temperature was removed, and only average air temperature was included. The odds of a strangles outbreak would be expected to increase by 3.5% for every 1.0 °C increase in average air temperature when minimum air temperature was held constant. In contrast, the odds of an outbreak would be expected to decrease by 3.1% for every 1.0 °C increase in minimum air temperature when average air temperature was held constant. Bootstrap resampling produced similar odds ratios and confidence intervals as the random remodeling and original logistic regression model for both minimum air temperature and average air temperature (Table 2). Random remodeling suggested that minimum air temperature was a significant predictor of outbreak in 39% of the models, while average air temperature was a significant predictor of outbreak in 58% of the models when α = 0.05 (Table 2). The proportion of models that included minimum air temperature increased to 68% and average air temperature increased to 78% when considering α = 0.10. Average AUC scores (0.515 when α = 0.05 and 0.526 when α = 0.10) suggested that model performance (e.g., predictive ability) was low and indicates this model would not properly discriminate outbreaks from non-outbreaks using these weather data.

## 4. Discussion

Our analyses suggest that the occurrence of strangles outbreaks can be affected by changes in temperature, though not precipitation (Table 1). The collected data show that the highest and lowest number of individual outbreaks occur during seasonal changes of the year, more specifically from winter into spring, when the highest number of cases were reported, and from summer into fall, when case numbers were at their lowest (Figure 2). Infection numbers appear to increase as temperatures increase through March, April and May and then drop when temperatures reach their peak during the summer months of June, July and August. These numbers then reach their maximal lowest in September and rise slightly before stabilizing during the winter months of December, January, and February. Though our model showed no significant correlation between maximum temperature and disease cases, it did show that the month-to-month change in average minimum temperature and the overall average temperature have significant impacts on the chances of outbreak occurrence. Both the general trends observed in case numbers and our logistic model lend support to the conclusion that changing temperatures may impact the chance of a strangles outbreak. The odds ratio of the overall average temperature variable suggests that for a 1 °C increase in temperature, the chance of an outbreak increases by 4% (Table 2). As overall average temperature can change by seven degrees from one month to the next and by 13 degrees over a season, as observed in this study, these fluctuations may result in the odds of an outbreak changing by 28% from month to month and as much as 52% across a season. Additionally, temperature fluctuations, and thereby increased outbreak risk, are likely to increase over time due to anthropogenic climate change.

Studies suggest that SEE, the bacterial causative agent of strangles, is spread by both direct and indirect contact [3,5] and our analyses suggest those mechanisms may be affected by the weather conditions. According to the Florida Department of Agriculture and Consumer Services, most horse-related events occur in the relatively warm summer months [25]. Before these events take place across the country, in the spring months, horses are often moved and trained to prepare for competitions. This would increase horses’ interactions with people, fomites, and other horses that are experiencing a higher risk of contracting infectious illnesses, perhaps leading to the increase in case numbers recorded during the April–May period. Changes to horse routines, environments, and increased exposure to other horses would directly increase the chance of a horse contracting strangles. While strangles is not a mandatory reportable disease, some states or equine competitions have vaccine requirements or disease screening alternatives for horses entering the state or competition [25]. This could explain why strangles outbreaks decrease during the summer months, as more mandatory testing is occurring for equine diseases than during other times of the year (Figure 2). Additionally, during this time, horses are more likely to be outdoors, and it has been shown that the bacterial causative agent of strangles is typically inhibited by warmer temperatures, as likely seen during the summer months. The slow increase in case numbers during the fall/winter months could be a result of horses being stabled for longer times during colder temperatures, when SEE has been shown to survive well in the external environment [6,7]. It has been shown that strangles is not the only equine disease to have the highest rate of infection during the spring season. Vector-borne equine disease studies have shown that spring months have a higher risk of disease spread because of vector movement brought on by warmer temperatures [26,27]. Our study is the first to suggest a relationship between disease risk with temperature that is strictly veterinary and not vector-borne.

Data acquisition for this study was difficult because veterinary diseases are particularly challenging to track, especially diseases affecting non-livestock animals, as very few are mandatory reportable diseases and the process for recording these diseases varies from state to state. As Figure 1 demonstrates, many states keep no records of diagnosed strangles cases. The majority of the reported data in this study was generously and voluntarily provided by veterinarians at veterinary diagnostic labs, professors maintaining university databases, government agencies in states that track cases, and through access to data found on the EDCC (Equine Disease Communication Center) website. Despite strangles being added to the NLRAD in 2017 [7], most state veterinary officials reported that their state did not track or keep any record of strangles. The lack of surveillance of strangles and other veterinary diseases creates challenges in fully understanding the impact of these pathogens on animals and animal owners while also impeding epidemiological research on their prevalence. As climate change continues, summer and winter temperatures in North America will rise and the frequency of extreme heat events will increase dramatically [28]. The impacts of these warmer conditions on strangles is uncertain and is likely to vary at local levels and can only be assessed with increased surveillance efforts.

In conclusion, this preliminary study sought to explore potential patterns or trends that might exist between cases of the equine disease strangles and weather parameters, specifically temperature and precipitation. This is the first study that has investigated how common environmental parameters may affect a non-vector-borne disease of veterinary importance in the USA. Our analyses found that while precipitation was not a significant factor in strangles outbreak occurrences, temperature could play a role in the incidence of this disease, especially the change in temperature associated with the seasons. While a significant logistic model was detected, the predictive ability of the model was low overall, which suggests this study should be considered more exploratory and further research is warranted in this area. More specifically, additional analyses that included weather data from smaller geographic regions or individual states would help better resolve potential trends in strangles outbreaks within the continental USA.

## Figures and Tables

**Table 1:** Results of a logistic regression of outbreak occurrence against monthly average precipitation (Precip_avg), minimum air temperature (Temp_min), maximum air temperature (Temp_max), and average air temperature (Temp_avg). Reported variables include model coefficient (Coeff; log odds), standard error (SE), odds ratio (OR) with 95% confidence interval (CI), *p*-value (*p*) assuming α = 0.05, and Akaike information selection criteria (AIC). Significant variables are in bold.

#	Model	Variable	Coeff	SE	OR	OR (95% CI)		*p*	AIC
						Lower	Upper		
1	Monthly Data	(Intercept)	−0.174	0.383	0.840	0.396	1.780	0.649	1675.0
		Precip_avg	0.001	0.001	1.001	0.999	1.003	0.277	
		Temp_min	1.475	7.123	4.372	3.759 × 10^−6^	5.125 × 10^6^	0.836	
		Temp_max	1.455	7.122	4.286	3.695 × 10^−6^	5.010 × 10^6^	0.838	
		Temp_avg	−2.934	14.243	0.053	3.896 × 10^−14^	7.144 × 10^10^	0.837	
2	Rel to 5y_avg	(Intercept)	0.023	0.124	1.023	0.802	1.307	0.852	1670.1
		Precip_5yr	0.002	0.002	1.002	0.999	1.006	0.119	
		Tmin_5yr	0.000	0.006	1.000	0.988	1.011	0.960	
		Tmax_5yr	−0.015	0.121	0.985	0.777	1.248	0.899	
		Tavg_5yr	−0.060	0.122	0.941	0.741	1.195	0.619	
3	Mon to Mon ∆	(Intercept)	0.015	0.059	1.015	0.904	1.140	0.802	1667.8
		Precip_m2m	0.001	0.001	1.001	0.999	1.003	0.212	
		Tmax_m2m	0.049	0.036	1.050	0.978	1.127	0.176	
		Tmin_m2m	−0.085	0.041	0.919	0.847	0.995	**0.039**	
		Tavg_m2m	0.033	0.015	1.033	1.003	1.064	**0.030**	
4	Mon to Mon ∆s	(Intercept)	0.011	0.059	1.011	0.901	1.136	0.849	1666.3
		Tmin_m2m	−0.031	0.016	0.969	0.939	1.000	**0.048**	
		Tavg_m2m	0.035	0.015	1.035	1.006	1.066	**0.018**	

Note: The first logistic regression included average weather data (Monthly Data), the second included how each variable compared to the 5-year average (2018–2022; Rel to 5yr_avg), the third assessed the change in each variable compared to the previous month (Mon to Mon ∆), and the last regression resulted from stepwise variable selection that started or ended with the same variable set as the third model (Mon to Mon ∆s).

**Table 2:** Results of performance tests including random remodeling (1) and bootstrap resampling (2) of significant logistic regression of outbreak occurrence against month-to-month change in minimum (Tmin_m2m) and average air temperature (Tavg_m2m). Coefficient estimates (Coeff; log odds) and odds ratio (OR) with 95% lower and upper confidence intervals (CI) are presented for both tests. The percentage of significant models (%) and average area under the curve (AUC) based on α = 0.05 (A5) and 0.10 (A10) was only calculated for significant models detected during the random remodel test.

No	Variable	Coeff	OR	Lower	Upper	%_A5_	%_A10_	AUC_A5_	AUC_A10_
1	(Intercept)	0.011	1.012	0.889	1.152	ns	ns	0.515	0.526
	Tmin_m2m	−0.036	0.965	0.931	0.999	39	68		
	Tavg_m2m	0.040	1.041	1.008	1.075	58	78		
2	(Intercept)	0.011	1.011	0.897	1.136	---	---	---	---
	Tmin_m2m	−0.031	0.969	0.941	0.999	---			
	Tavg_m2m	0.035	1.035	1.007	1.066	---			

Note: Some variables were not significant (ns) for any of the 100 individual logistic models tested. Significance of individual models and average AUC was not determined for the bootstrap resampling because model parameters represent averages across all 1000 iterations, which are indicated by the dashed (---) line above.

## Data Availability

Outbreak and weather data used in this analysis have been uploaded as a Supplementary File (.xlsx).

## Supplementary Materials

The following supporting information can be downloaded at https://www.mdpi.com/article/10.3390/pathogens12091106/s1. Figure S1: Average five-year precipitation by month; Table S1: Number of reported strangles cases by state; Table S2: Outbreak and weather data.
